# A dual-fluorescence approach for turn-on ammonia and turn-off explosive picric acid detection *via* ESIPT inhibition: experimental, theoretical, and biological studies

**DOI:** 10.1039/d5ra05068e

**Published:** 2025-09-11

**Authors:** Malavika S. Kumar, Avijit Kumar Das

**Affiliations:** a Department of Chemistry, Christ University Hosur Road Bangalore Karnataka 560029 India avijitkumar.das@christuniversity.in

## Abstract

A fluorescent naphthalene–anthracene dyad (AMN) was developed as a dual-mode sensor for turn-on detection of ammonia (NH_3_) and turn-off detection of picric acid (PA). AMN initially emits strong fluorescence at 427 nm due to excited-state intramolecular proton transfer (ESIPT), showing a large 62 nm Stokes shift. Upon PA addition, fluorescence is quenched and red-shifted to 463 nm. Conversely, NH_3_ induces a red shift to 435 nm. These spectral responses are attributed to ESIPT inhibition *via* strong hydrogen bonding between the hydroxyl group of AMN and the analytes. AMN has been successfully applied in dipstick-based PA detection and as a low-cost food spoilage indicator for NH_3_. Detection limits are 8.77 μM for PA and 5.29 μM for NH_3_, with a Stern–Volmer constant of 5.62 × 10^5^ M^−1^ for picric acid. Additionally, AMN shows ratiometric fluorescence upon interaction with BSA and ct DNA, accompanied by notable absorption changes. These findings, supported by UV-vis, fluorescence spectroscopy, NMR, molecular docking, and DFT studies, underscore the potential of AMN as a multifunctional fluorescent sensor for environmental and biological applications.

## Abstract 2

A fluorescent naphthalene–anthracene dyad (AMN) was developed as a dual-mode sensor for turn-on detection of ammonia (NH_3_) and turn-off detection of picric acid (PA).

## Graphical abstract

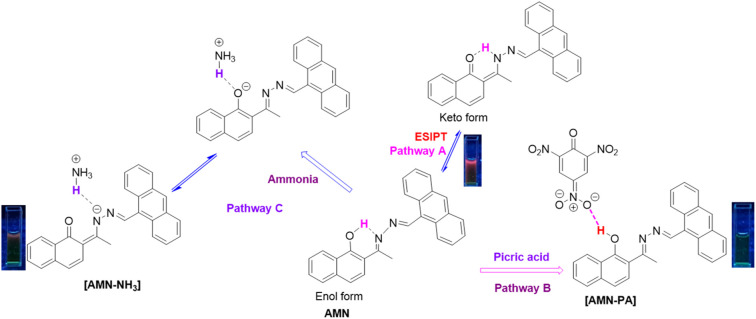

## 1. Introduction

Recently, organized terror attacks all around the world have compelled scientists to develop effective techniques for detecting explosives. Nitro compounds constitute a significant percentage of common explosives. TNT was the most broadly used nitroaromatic compound (NAC) prior to World War I.^1–3^ However, ascribed to its strong explosive force, picric acid (PA) has surfaced as an ideal substitute and has been employed as a raw material for lethal weapons.^4,5^ It is extensively utilized in the pharmaceutical, dye, and rocket fuel industries.^6–9^ The intake of PA can lead to anemia, cancer, cyanosis, liver problems, and skin and eye irritation.^10–14^ Therefore, effectively monitoring and detecting trace amounts of PA in both vapor and solution phases is crucial for ensuring social and environmental safety.

On the other hand, several types of gas sensors have been designed and utilized for precarious gas sensing in various fields, including self-propelled manufacturing, ecological investigation, air quality control, therapeutic applications, and more. NH_3_ (ammonia) is one of the most common caustic gases in the biosphere and is released through both industrial and natural activities.^15,16^ In nature's nitrogen cycle, NH_3_ is produced by the breakdown of organic nitrogen molecules in plants, human and animal waste, and the microbial decomposition of sewage.^17–19^ Industrial processes such as metallurgical operations, mining, ceramic manufacturing, the synthesis of other chemical compounds, agricultural practices, and the use of domestic cleaning products also contribute to the artificial production of NH_3_. Chronic exposure to NH_3_ vapors can cause gastrointestinal illnesses, kidney problems, and nasal erosion ulcers.^20–22^ Therefore, there is a critical need to develop effective sensor systems for the selective detection and monitoring of trace concentrations of NH_3_ in the gas phase or NH_4_^+^ in the solution phase.

**Scheme 1:**
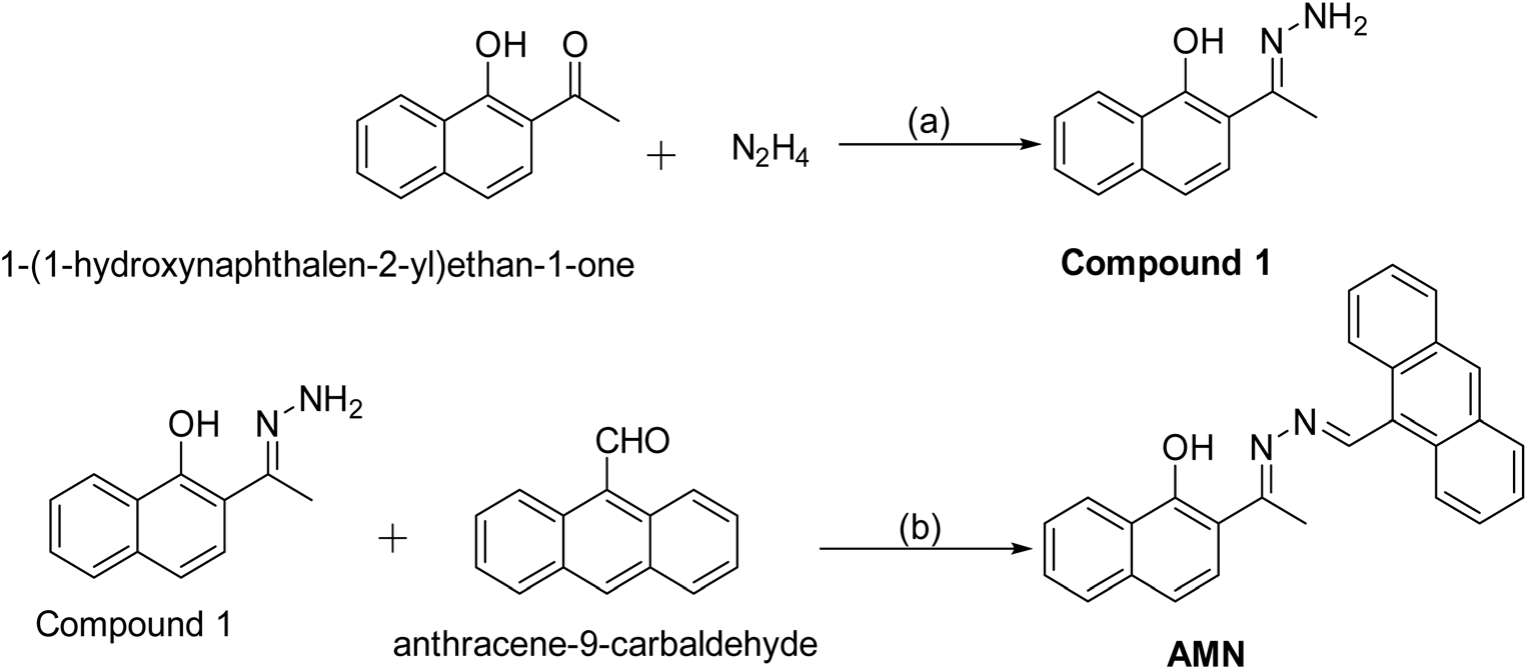
Synthesis of AMN: (a) EtOH, rt, 24 h. (b) EtOH, rt, 48 h.

**Scheme 2:**
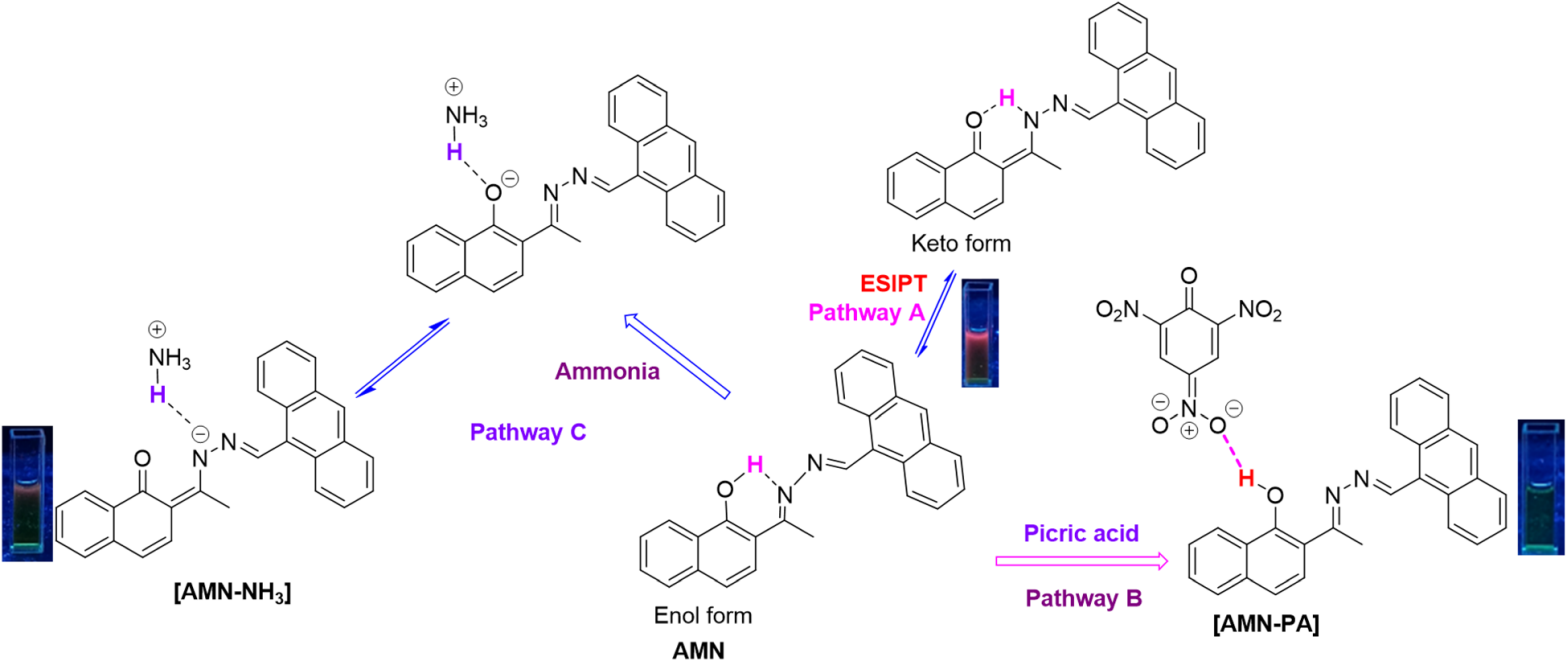
Probable binding mechanism of AMN with PA and NH_3_.

Recently, countless instrumentation techniques have become available for the detection and quantification of various analytes, including spectrophotometry, gas chromatography–mass spectrometry (GC–MS), flow spectroscopy, potentiometric electrodes, and infrared absorption. Electrochemical sensing is another widely used technology due to its high sensitivity and cost-effectiveness.^22–28^ However, the aforementioned instrumentation techniques have limitations in practical usage for continuous analyte monitoring, as they often involve chemical usage, require extensive pre-treatment, depend on expensive apparatus, and are not advisable for on-site real-time monitoring.^29,30^ To overcome these drawbacks, optical methods have emerged in recent years as efficient and practical solutions for sensing analytes under ambient conditions. Among these, fluorescence-based sensing techniques have gathered significant research interest due to their high detection sensitivity, low instrument cost, fast response time, and ease of use. Fluorescence-based sensing is particularly effective for detecting target analytes with high sensitivity.^31^ In this respect, ESIPT (excited-state intramolecular proton transfer) phenomenon, a photo-induced proton transfer *via* an intramolecular hydrogen bond, is highly valued in organic optoelectronic materials for its photochemical and photophysical applications.^32^ It occurs in molecules with intramolecular hydrogen bonding between donors and acceptors, involving rapid enol-to-keto phototautomerization (>10^12^ s^−1^), followed by radiative decay and reverse proton transfer (RPT) to restore the enol form (Scheme 2, pathway A). Its large Stokes shift enhances efficiency and minimizes self-absorption.^33^ Common ESIPT fluorophores include derivatives of 2-(2′-hydroxyphenyl)benzimidazole, benzoxazole, benzothiazole, quinoline, benzophenones, flavones, anthraquinones, and benzotriazoles.^34^ Herein, we have developed a ESIPT based Schiff base probe 2-((E)-1-(((Z)-anthracen-9-ylmethylene)hydrazineylidene)ethyl)naphthalen-1-ol (AMN), for selective detection of picric acid and ammonia. The synthesis of AMN involved two steps involving reaction of 1-(1-hydroxynaphthalen-2-yl)ethan-1-one with hydrazine to produce compound 1 followed by its reaction with anthracene-9-carbaldehyde in ethanol to yield AMN (Scheme 1). The chemical structures of compound 1 and AMN were characterized using mass spectra and NMR analysis (Fig. S8–S10). Additionally, DNA and BSA binding studies were conducted to explore its potential biological applications. The interaction of Schiff-base ligands like AMN with DNA and BSA molecules has been investigated experimentally and theoretically in order to formulate new pharmacological medicines.^35,36^ The amino acid residues can readily bind with drug molecules due to a large number of binding sites, which increases solubility and decreases toxicity.^37^ Similarly, a drug molecule can attach to DNA by major or minor groove binding, hydrogen bonding/electrostatic interactions, or intercalation. Therefore, it is essential to comprehend how drugs interact with biomolecules in order to properly describe the pharmacokinetic profile and design of drugs.^38–41^ For practical purposes, a low-cost food spoilage indicator was developed for NH_3_ sensing, along with a dipstick method for the detection of picric acid.

## 2. Experimental

### 2.1 Materials and instrumentation

Sigma-Aldrich Chemicals Private Limited supplied the chemicals and solvents, which were used without any extra purification, unless stated otherwise. Melting points were found using an open-mouth capillary and a hot-plate melting point device. A Brucker 400 MHz device was employed to record ^1^H-NMR spectra. DMSO-d_6_ was utilized as the solvent and TMS as the internal standard for the NMR spectra. Chemical shifts are displayed in *δ*-units and the ^1^H–^1^H coupling constants are expressed in Hz. A PerkinElmer Lambda 30 UV-vis titration device was utilised for the UV-vis titration experiments, whereas a Shimadzu RF-5301 PC was utilised for the fluorescence experiment. A fluorescent cell with a 10-mm path was used in a fluorescence spectrofluorometer.

### 2.2 Synthesis and characterizations

#### 2.2.1 Synthesis of compound 1

In a 100 mL round-bottom flask, 1-(1-hydroxynaphthalen-2-yl)-ethan-1-one (100 mg, 0.36 mmol) was dissolved in 10 mL of ethanol, followed by the dropwise addition of hydrazine. The reaction mixture was stirred at room temperature for 24 hours, resulting in the precipitation of compound 1. The precipitate was then filtered and washed sequentially with ethanol and ether to obtain a solid yellow product. (Yield: 80 mg, 75%).

^1^H NMR (DMSO-d_6_, 400 MHz): 14.91 (s, ^1^H, –OH), 10.05 (s, ^1^H), 8.21 (d, ^1^H, *J* = 7.6 Hz), 7.79 (t, ^1^H, *J* = 8.8 Hz), 7.58 (d, ^1^H, *J* = 8.8 Hz), 7.46 (m, 2H), 7.32 (d, 2H, *J* = 8.8 Hz), 6.61 (s, 2H), 2.25 (s, 3H).

#### 2.2.2 Synthesis of AMN

Compound 1 (100 mg, 0.5 mmol) was reacted with anthracene-9-carbaldehyde (103 mg, 0.5 mmol) in 12 mL of ethanol in a 100 mL round-bottom flask for 48 hours at room temperature. The reaction progress was monitored by TLC, and an orange solid precipitate was formed. Upon completion of the reaction, the precipitate was filtered, washed with ethanol and diethyl ether followed by vacuum-dried to obtain an orange solid.

(Yield: 150 mg, 77%). Mp: 200–210 °C. ^1^H NMR (DMSO-d_6_, 400 MHz): 15.35 (s, ^1^H, –OH), 10.05 (s, ^1^H), 8.89 (t, 2H, *J* = 15.6 Hz), 8.41 (d, ^1^H, *J* = 8 Hz), 8.21 (d, ^1^H, *J* = 8.4 Hz), 7.91 (d, 2H, *J* = 8 Hz), 7.86 (d, ^1^H, *J* = 8.8 Hz), 7.72 (d, ^1^H, *J* = 6.8 Hz), 7.68 (d, 2H, *J* = 6 Hz), 7.64 (d, 3H, *J* = 7.2 Hz), 7.60 (d, ^1^H, *J* = 7.6 Hz), 7.47 (d, ^1^H, *J* = 8.8 Hz), 2.88 (s, 3H). Mass (*m*/*z*, %): M^+^ calculated for chemical formula: C_27_H_20_N_2_O is 388.16; found: 389.95 (M + H)^+^.

## 3. Results and discussion

### 3.1 Spectroscopic response of probe AMN toward NH_3_ and PA

**Fig. 1:**
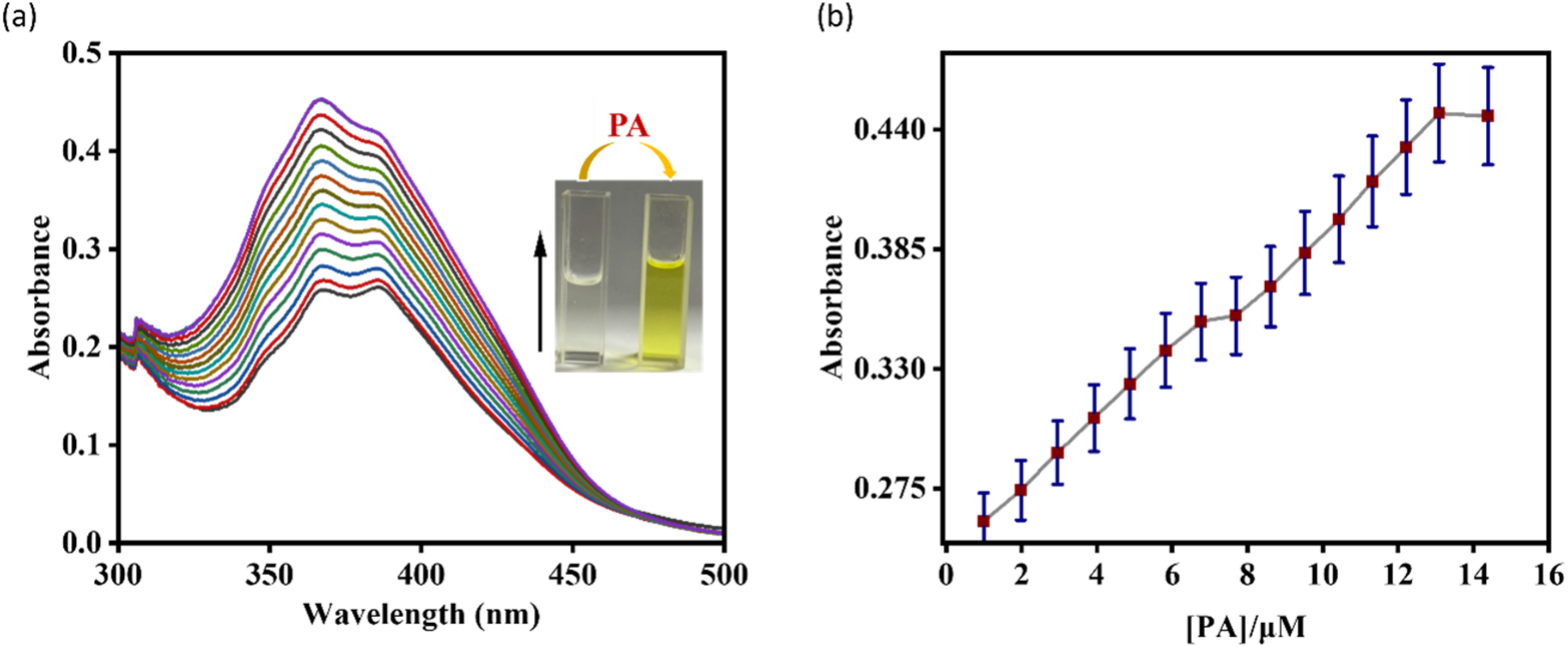
(a) UV-vis titration spectra of AMN (*c* = 20 μM) with picric acid (*c* = 200 μM). (b) Absorbance changes of AMN as a function of picric acid concentration (error quantity, 5%; *Y* error bar for both [±] deviation).

The spectrophotometric and spectrofluorometric titration of AMN with NH_3_ and PA were conducted in CH_3_CN/HEPES buffer (7 : 3, v/v, pH 7.4). The stability of the fluorescence of AMN is almost same in different ratios of CH_3_CN/HEPES buffer mixtures and the spectrofluorimetric titration showed comparable emission changes (Fig. S14). Initially, the probe AMN showed a weak absorption peak at 365 nm, but with incremental addition of picric acid, the absorption band at 365 nm increased gradually in a dose dependent manner and naked eye color changes from colorless to pale yellow (Fig. 1). In case of spectrophotometric study of AMN in presence of NH_3_, there is a slight decrease of absorbance at 365 nm (Fig. S15).

**Fig. 2:**
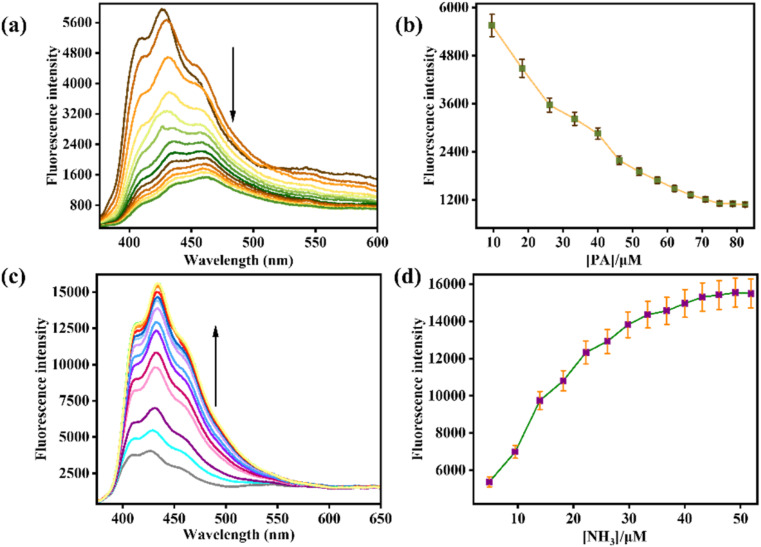
(a) Fluorescence titration spectra of AMN (*c* = 20 μM) with PA (*c* = 200 μM). (b) The changes of emission intensity with variation of PA concentration along with error bars (error amount, 5%; *Y* error bar for both [±] deviation). (c) Fluorescence titration spectra of AMN (*c* = 20 μM) with NH_3_ (*c* = 200 μM). (d) The changes of emission intensity with variation of NH_3_ concentration along with error bars (error amount, 5%; *Y* error bar for both [±] deviation).

In the fluorescence spectroscopy, the sensing capabilities of AMN towards various analytes were examined in a CH_3_CN/HEPES buffer mixture (7 : 3, v/v, pH 7.4) at an excitation wavelength of 364 nm. Primarily, AMN exhibited a strong emission band at 427 nm. However, upon the gradual incremental addition of PA, the emission intensity got reduced by 3-fold, accompanied by the appearance of a red shifted emission band at 463 nm (Δ*λ* = 36 nm) (Fig. 2a).

Conversely, when NH_3_ was incrementally introduced in the solution of AMN, a fluorescence turn-on with 3-fold intensity increase was observed. The progressive addition of NH_3_ led to a gradual increment in the emission band at 427 nm, with the appearance of a new red-shifted emission band emerged at 435 nm (Δ*λ* = 8 nm) (Fig. 2c). Binding isotherm of AMN with PA and NH_3_ has been presented at 427 nm and 432 nm respectively (Fig. 2b and d).

The limit of detections of AMN towards PA and NH_3_ have been calculated to be 8.77 μM and 5.29 μM, respectively, using the formula DL = *K* × Sb_1_/*S*, in which S is the calibration curve's slope and Sb_1_ is the blank solution's standard deviation (Fig. S1 and S2).^42^ Based on the first order rate equation and the variations of fluorescence intensity of AMN caused by the addition of PA and NH_3_ at various time intervals, the rate constants were determined to be 143.24 s^−1^ and 330.41 s^−1^ respectively demonstrating the fast response of AMN towards PA and NH_3_ (Fig. S5 and S6). The Stern–Volmer quenching constant (*K*_SV_) was calculated to assess quenching efficiency and sensitivity. For PA the predicted the quenching constant (*K*_SV_) values are 5.62 ×10^5^ M^−1^ (Fig. S7). This high quenching constant value indicates that PA have very good fluorescence quenching abilities towards AMN.

### 3.2 Interference study

**Fig. 3:**
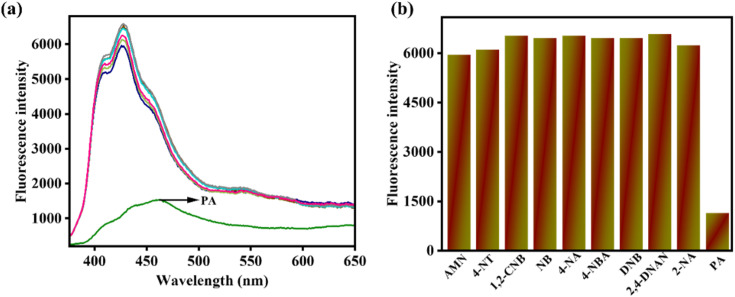
(a) Comparison fluorescence spectra of AMN (*c* = 20 μM) upon addition of different guest analytes (*c* = 200 μM) (15 equiv.) at excitation at 364 nm. (b) Comparison fluorescence spectra of AMN upon addition of different guest analytes in bar diagram.

To evaluate the selectivity of AMN towards PA, fluorescence experiments were conducted with AMN in the presence of various interfering nitroaromatic compounds in CH_3_CN/HEPES buffer mixture (7 : 3 v/v, pH 7.4). Notably, upon the addition of PA to the receptor solution, the fluorescence intensity was significantly quenched, leading to a diminished emission signal at 427 nm. However, when other interfering nitroaromatic compounds such as dinitrobenzene (DNB), nitrobenzene (NB), 4-nitrotoluene (4-NT), 4-nitroaniline (4-NA), 4-nitrobenzoic acid (4-NBA), 1-chloro-2-nitrobenzene (1,2-CNB), and 2,4-dinitroanisole (2,4-DNAN) were introduced, no discernible fluorescence changes were observed (Fig. 3a).

This indicates that AMN exhibits high selectivity for PA. In the bar representation, the brown bar with lower intensity represents the fluorescence signal response of AMN with PA, while other brown bars with higher intensities indicate no significant interference in fluorescence of AMN with other interfering analytes (Fig. 3b).

**Fig. 4:**
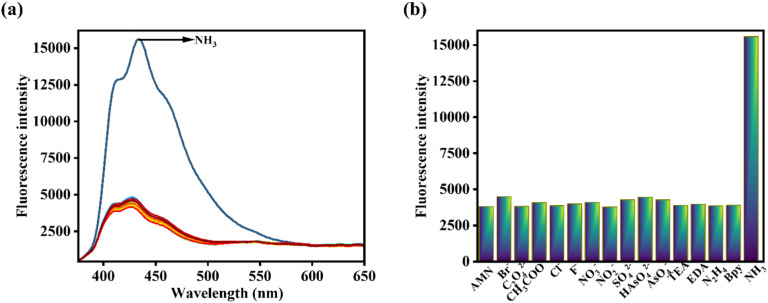
(a) Comparison fluorescence spectra of AMN (*c* = 20 μM) upon addition of different guest analytes (*c* = 200 μM) (15 equiv.) at excitation at 364 nm. (b) Comparison fluorescence spectra of AMN upon addition of various different analytes in bar diagram.

Alternatively, the selectivity of AMN towards NH_3_ has been justified by conducting a fluorescence experiment with various interfering anions like Cl^−^, CH_3_COO^−^, Br^−^, F^−^, NO_2_^−^, C_2_O_4_^−^, SO_4_^2−^, H_2_O_2_, NO_3_^−^, OCl^−^, triethylamine (TEA), ethylenediamine (EDA), N_2_H_4,_ piperidine (bpy) in CH_3_CN : HEPES buffer (7 : 3, v/v, pH 7.4). Significantly, the presence of NH_3_ in the AMN solution led to the appearance of a turn-on fluorescence response. However, upon the addition of all interfering analytes, the emission intensity at 427 nm remained unchanged. This finding demonstrates the remarkable selectivity of AMN for NH_3_ (Fig. 4a). In the bar representation, the green bar with lower intensity indicates the fluorescence signal response of AMN with NH_3_, while other blue bars with higher intensities indicate no notable interference in fluorescence of AMN with other interfering analytes (Fig. 4b).

### 3.3 Plausible binding mechanism of AMN with picric acid and ammonia

To elucidate the binding of AMN with picric acid and ammonia, UV-vis, fluorescence and ^1^H NMR studies were performed in DMSO-d_6_. In the absence of analytes, the ligand AMN exhibits strong orange fluorescence due to the conversion of the enol intermediate to the keto form through an ultrafast photo-induced tautomerization process *via* excited-state intramolecular proton transfer (ESIPT). This process involves the transfer of a proton from the acidic hydroxyl group to the basic imine nitrogen, facilitated by the formation of a six-membered transition state (pathway A, Scheme 2). Upon exciting at 364 nm, AMN exhibits strong naked eye orange fluorescence in solid state with an intense emission signal at 629 nm (Fig. S16). In solution phase, AMN displays a strong absorbance signal at 365 nm and an emission signal at 427 nm, with a large Stokes shift of approximately 62 nm, which indicates the occurrence of the ESIPT phenomenon within the molecule.^43^ However, the changes in absorption and fluorescence upon exposure to picric acid and ammonia are attributed to the suppression of the ESIPT process due to the strong interactions between the hydroxyl group of AMN with ammonia and picric acid.

Picric acid is a strong acid due to the high stability of its conjugate base, which is stabilized by extensive delocalization of the negative charge across the three nitro groups. Although picric acid can serve as both a hydrogen bond donor and acceptor, its conjugate base arises from proton loss and is strongly stabilized by the electron-withdrawing nitro substituents. The hydroxyl (–OH) group enables hydrogen bond donation, while the nitro oxygen atoms facilitate hydrogen bond acceptance, a property that is effectively exploited in the detection of picric acid.^44^ Therefore, in the presence of picric acid, AMN exhibited enhanced absorbance and decreased fluorescence, attributed to the strong hydrogen bonding interactions between the nitro groups of picric acid and the hydroxyl group of AMN (pathway B, Scheme 2). This interaction was further supported by ^1^H NMR experiments and theoretical studies. In the ^1^H NMR experiments due to this strong interaction, the addition of PA to a solution of AMN resulted in a significant downfield shift of the phenolic hydroxyl proton signal and the decrease in electron density due to hydrogen bonding caused extensive broadening of the hydroxyl proton signal, shifting it from *δ* 15.25 ppm to *δ* 15.55 ppm. Additionally, other aromatic proton signals exhibited slight downfield shifts due to the formation of the AMN-PA charge transfer complex (Fig. S11).

On the other hand, AMN displayed turn on fluorescence response due to the strong basicity of ammonia, which led to the deprotonation of the hydroxyl group, forming a 1-naphthoxide ion stabilized by resonance through the imine moiety (pathway C, Scheme 2). This deprotonation was validated by ^1^H NMR studies, where, in the presence of NH_3_, the hydroxyl proton signal at *δ* 15.25 ppm was almost completely diminished, while other aromatic proton signals shifted downfield, exhibiting higher *δ* values (Fig. S12).

### 3.4 Theoretical study

**Fig. 5:**
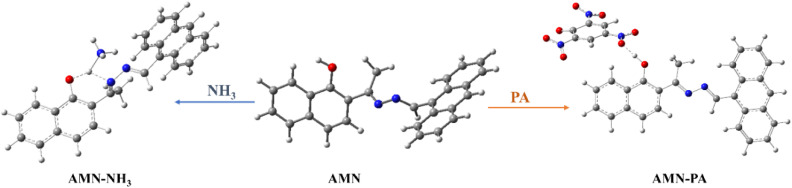
Geometry optimized molecular structures of AMN, AMN-NH_3_ complex and AMN-PA complex.

**Fig. 6:**
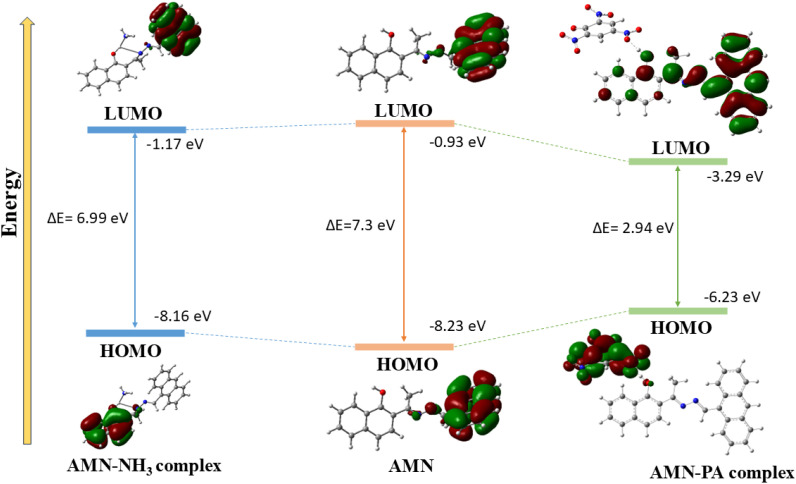
Frontier molecular orbital with energy difference of AMN, AMN-PA complex and AMN-NH_3_ complex.

To explain the interactions mechanism between AMN with NH_3_ and PA, we have performed structure optimization of AMN and AMN-PA, AMN-NH_3_ complexes using DFT calculations with B3LYP/6-31G (d,p) level of theory in the Gaussian 09 W software package (Experimental details in SI). The optimization of the ligand AMN and its complexes was carried out by generating a starting model based on the DFT-optimized structures of AMN, AMN-PA, and AMN-NH_3_ complexes (Fig. 5). Consistent with ^1^H NMR observations, a strong hydrogen bonding interaction was identified between the hydroxyl group of AMN and the nitro group of PA. The AMN exhibited a notable energy gap of 7.3 eV between HOMO (−8.23 eV) and LUMO (−0.93 eV). However, upon interaction with PA and NH_3_, the energy gaps were reduced to 2.94 eV (HOMO: −6.23 eV; LUMO: −3.29 eV) and 6.99 eV (HOMO: −8.16 eV; LUMO: −1.17 eV) respectively, indicating structural stabilization due to the strong interactions of AMN with PA and NH_3_ (Fig. 6). For chemosensor AMN, the frontier molecular orbitals (FMOs) were primarily distributed over the more electron-rich anthracene moiety, resulting in only a modest intramolecular charge transfer (ICT) character. However, in the AMN-PA complex, a significant spatial separation of FMOs was observed. The HOMOs were mainly localized on the electron-deficient picric acid moiety, while the LUMOs were predominantly distributed along the AMN ligand. Similarly, for AMN-NH_3_ complex, the HOMOs were mainly localized on the naphthalene ring, while the LUMOs were predominantly distributed along the anthracene moiety of AMN ligand. This orbital distribution suggests strong interaction between AMN with PA and NH_3_ within the complex, which affects the orbital distribution of LUMO and HOMO asymmetrically, thereby influencing a notable optical response of AMN in presence of PA and NH_3_.

### 3.5 Biological applications

#### 3.5.1 DNA and protein binding studies

**Fig. 7:**
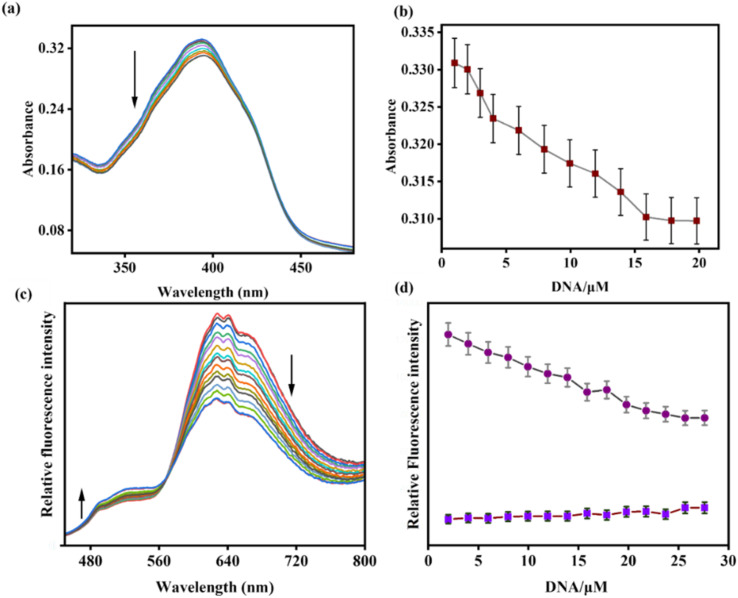
(a) UV-vis and (c) fluorescence titration spectra of AMN (*c* = 20 μM) upon incremental addition of ct DNA (*c* = 2 mM in basepair) in Tris–HCl buffer, pH = 7.2. Plot of ct DNA concentration *vs.* intensity in (b) UV-vis and (d) fluorescence titration spectra (error quantity, 1% and 5% respectively; *Y* error bar for both [±] deviation).

**Fig. 8:**
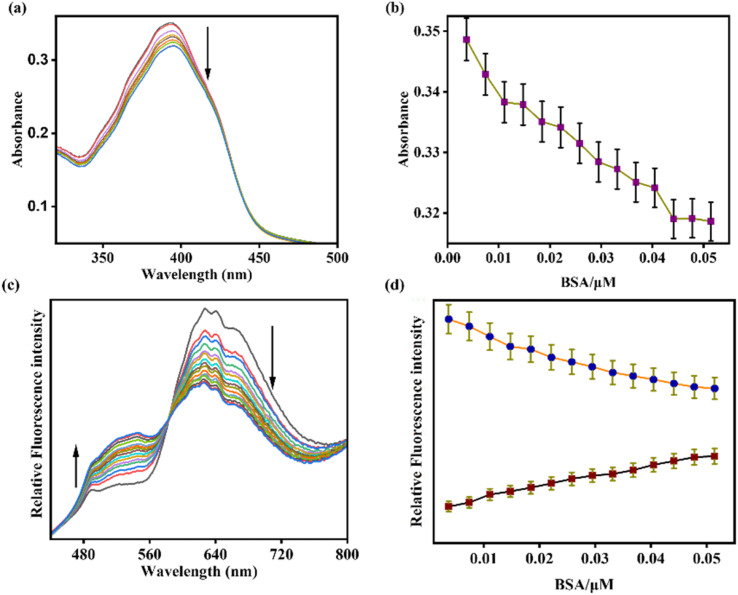
(a) UV-vis and (c) fluorescence titration spectra of AMN (*c* = 20 μM) upon incremental addition of BSA (*c* = 7.4 μM) in Tris–HCl buffer, pH = 7.2. Plot of BSA concentration *vs.* intensity in (b) UV-vis and (d) fluorescence titration spectra (error quantity, 1% and 5% respectively; *Y* error bar for both [±] deviation).

The binding properties of AMN with duplex ct DNA and Bovine Serum Albumin (BSA) were studied in Tris–HCl buffer (pH 7.2) using fluorescence and UV-vis spectral analysis. Initially, the ligand AMN exhibited a strong absorption signal at 395 nm. Upon increasing the concentration of BSA and ct DNA in the AMN solution, the absorption peak at 393 nm gradually decreased (Fig. 7a and 8a). Similarly, upon excitation at 418 nm, AMN showed a strong fluorescence with an emission signal at 625 nm. However, the incremental addition of ct DNA and BSA to the AMN solution led to a gradual decrement in fluorescence intensity at 625 nm with the appearance of a blue-shifted ratiometric fluorescence signal at 524 nm (Δ*λ* = 101 nm), with an isoemission point at 568 nm. Although the ratiometric response of AMN with ct DNA is lower as compared to BSA (Fig. 7c and 8c). The limit of detection (LOD) of AMN for ct DNA and BSA was calculated to be 3.48 μM and 5.17 μM, respectively (Fig. S3 and S4). Based on a non-linear fluorometric binding isotherm, the binding constants of AMN with ct DNA and BSA were found to be 5 × 10^4^ M^−1^ and 7.4 × 10^4^ M^−1^ respectively (Fig. S17).

In order to further investigate the interaction mode between ct DNA and AMN, the competition assay mainly by replacement of intercalating dye like ethidium bromide (EB) from ct DNA was employed. Herein, the EB replacement assay has been performed by the fluorescence experiment in Tris–HCl buffer, pH = 7.2. Initially, high fluorescence intensity has been observed for EB bound ct DNA at 625 nm, which was significantly enhanced with the increase in concentrations of the compound AMN. This fluorescence enhancement is mainly due to the replacement of EB from EB bound ct DNA complex by AMN. This confirms the intercalation binding mode of AMN with ct DNA (Fig. S13). The significant changes in absorbance and ratiometric emission response of AMN in the presence of ct DNA and protein BSA are attributed to restricted conformational mobility, which minimizes non-radiative deactivation of the excited state. This phenomenon is consistent with previous observations for several styryl- and stilbene-based dyes.^45^ Furthermore, the association of AMN is primarily driven by attractive dispersion forces, such as van der Waals interactions and π–π stacking, as well as the thermodynamically favorable release of counter-ions from DNA. These factors collectively restrict molecular flexibility and suppress the formation of ICT or CS excited states within the binding site.^46^ Additionally, the polar binding cavities of DNA and proteins effectively stabilize the CS or ICT states, leading to ratiometric fluorescence.

#### 3.5.2 *In silico* molecular docking studies

**Fig. 9:**
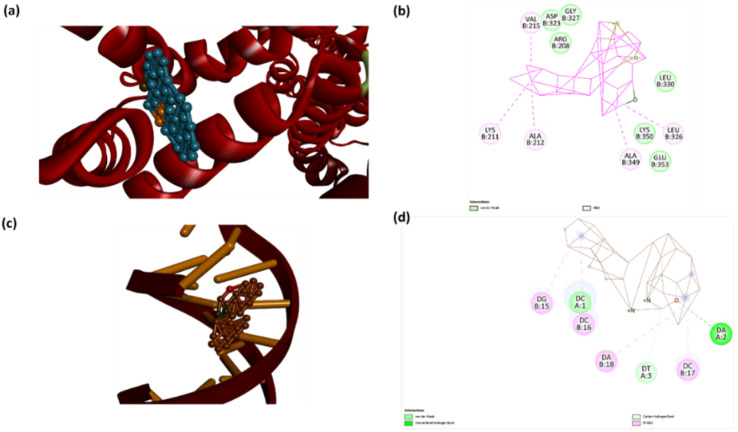
Possible binding mechanisms of AMN with BSA and ct DNA. Ribbon (a) and (c) views of the BSA and ct DNA complexes, respectively; (b) and (d) represents two-dimensional interaction of BSA and DNA respectively.

The binding interactions of AMN with ct DNA and BSA were further validated through molecular docking studies, a widely used method for speculating the binding affinity and interaction sites of biomolecules, including proteins and DNA. The docking simulations were performed using AutoDock Vina, which facilitated the analysis of the binding conformations of AMN with both BSA and ct DNA. Molecular docking studies revealed that AMN exhibited strong binding with the BSA protein through interactions with amino acid residues LYS211, ALA212, LEU326, ALA349, ASP323, GLY327, ARG208, LEU326, LYS350, and GLU353, with a binding affinity of −16.9 kcal mol^−1^ (Fig. 9a and b). Similarly, docking analysis of AMN with ct DNA indicated an intercalation binding mode, with a binding energy of −12.9 kcal mol^−1^ (Fig. 9c and d). The interaction between AMN and the DNA double helix was primarily established through deoxyribose guanine (15), deoxyribose thymine (3), deoxyribose cytosine (1, 16, 17), and deoxyribose adenine (2, 18) nucleosides (Fig. 9c and d). Overall, the docking studies suggest that AMN exhibits a strong binding affinity for both DNA and BSA, highlighting its potential as an effective biomolecular probe.

### 3.6 Practical application

#### 3.6.1 Dipstick method (PA) and low-cost food spoilage indicator (NH_3_)

**Fig. 10:**
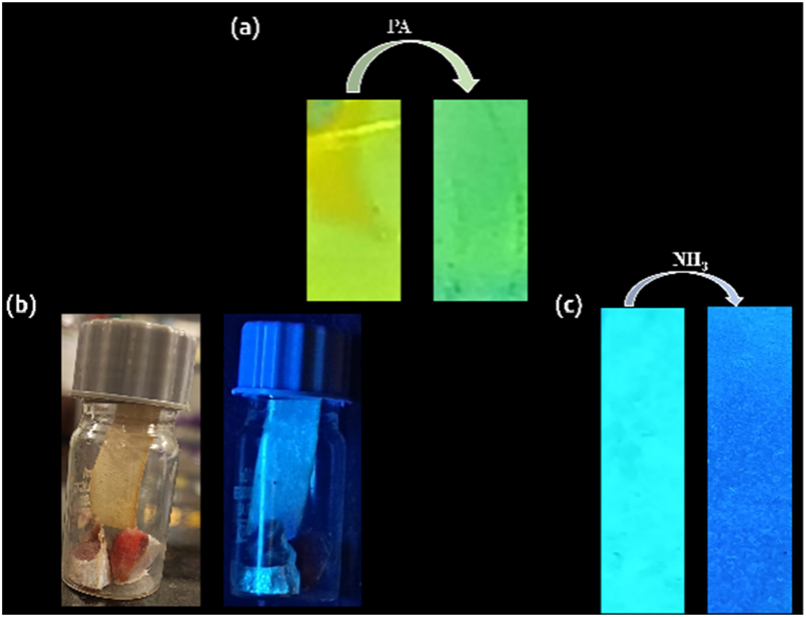
(a) Photograph of TLC plates with AMN itself (left) (*c* = 20 μM) and presence of PA (right) (*c* = 200 μM). (b) The experimental setup of a paper strip placed above fresh fish (left) (naked eye view) and above spoiled fish (right) (under UV chamber), in glass vials covered tightly with a lid. (c) AMN coated test strips before exposure to NH_3_ gas with fresh fish (left); after exposure to NH_3_ gas from spoiled fish (right) under UV lamp.

For the real-world application of the sensor AMN, dipstick method and low-cost food spoilage indicator experiments were performed for the detection of PA and NH_3_ respectively. To evaluate the effectiveness of the AMN sensor for detecting PA, a dipstick method was employed using test strips prepared by immersing TLC plates in the receptor solution (*c* = 20 μM). Under UV light, the ligand exhibited a strong fluorescence, which significantly diminished upon the addition of PA, confirming the quenching property of AMN towards PA (Fig. 10a). Such dipsticks or test strips are advantageous as they allow for the immediate acquisition of qualitative data without requiring instrumental analysis.

Additionally, various volatile amines and ammonia vapors are released during food spoilage.^47^ Freshly cut sardine fish were placed in glass bottles with AMN-coated filter sheets attached to the interior of the bottles to investigate the possible application of AMN for detecting biogenic volatile ammonia vapours (Fig. 10b). After three days of exposure to spoiled fish at 25 °C, the AMN-coated filter sheets were examined under 365 nm UV light. A significant enhancement in fluorescence intensity was observed, whereas in the absence of fish meat, the AMN-coated filter sheets retained the initial fluorescence (Fig. 10c). These results demonstrate that AMN exhibits high sensitivity and reactivity towards naturally generated ammonia vapors, enabling detection without the need for analytical instruments.

## 4. Conclusion

In conclusion, the naphthalene–anthracene dyad (AMN) has been demonstrated as a highly effective dual fluorescence-based sensing approach for the detection of ammonia and picric acid. The selective fluorescence responses—turn-on bathochromic shift for NH_3_ and turn-off quenching with a bathochromic shift for PA—enable easy visual and spectral detection of these analytes. The strong interactions between AMN with PA and NH_3_ inhibiting the ESIPT process underpin the sensing mechanism, validated by extensive spectroscopic and computational analyses. Moreover, AMN displays a ratiometric fluorescence response when binding with BSA protein and ct DNA, along with notable alterations in its absorption spectra and the ability of AMN to interact with biomolecules further expands its scope for biochemical applications. For the practical applications of the sensor AMN, dipstick method and low-cost food spoilage indicator experiments were utilized for the detection of PA and NH_3_ respectively without requiring any sophisticated instrumental analysis. Overall, AMN emerges as a promising and versatile fluorescence probe with significant potential for environmental and biomedical sensing.

## Conflicts of interest

There are no conflicts of interest to declare.

## Supplementary Material

RA-015-D5RA05068E-s001

## Data Availability

Supplementary information: The data supporting this article have been included as part of the SI. See DOI: https://doi.org/10.1039/d5ra05068e.
