# Supplementary material for: A dual-fluorescence approach for turn-on ammonia and turn-off explosive picric acid detection via ESIPT inhibition: experimental, theoretical, and biological studies
Source: RSC Adv. 2025 Sep 11;15(39):32894–905. doi: 10.1039/d5ra05068e (PMC12423931; doi:10.1039/d5ra05068e)
Supplement: RA-015-D5RA05068E-s001 [file RA-015-D5RA05068E-s001.pdf]

Supplementary Information (SI) for RSC Advances.
This journal is © The Royal Society of Chemistry 2025

A Dual-Fluorescence Approach for Turn-On Ammonia and Turn-Off Explosive Picric Acid Detection via ESIPT Inhibition: Experimental, Theoretical, and Biological Studies

Malavika S Kumar and Avijit Kumar Das*

Department of Chemistry, Christ University, Hosur Road, Bangalore, Karnataka, 560029 India. Email: avijitkumar.das@christuniversity.in

CONTENTS

1. **General methods of UV-vis and fluorescence titration experiments ……………….2**

2. **Determination of fluorescence quantum yield……………..…………………..........2**

3. **Calculation of the detection limit…………………………………………………..2-4**

4. **Rate constant calculation…………………………………………………………..4**

5. **Stern Volmer plot ………………………………………………………………….5**

6. **^{1}H NMR and Mass spectrum of AMN…………………………………………….5-6**

8. **^{1}H NMR spectra of AMN with picric acid and NH_3…………………………………7**

9. **Ethidium Bromide (EB) competition assay …………………………………………8**

10. **Solid state fluorescence and binding constants with ct DNA and BSA……………8**

11. **Methods for Computational details and silico molecular docking studies…………9**

12. **Comparison table of reported sensors for picric acid………………………………10**

13. **References………………………………………………………………………..11**

General method of UV-vis and fluorescence titration:

By UV-vis method:

For UV-vis titrations, stock solution of the sensor was prepared ($c = 2 \times 10^{-5}$ M) in CH_3CN-HEPES buffer (7/3, v/v, 25ºC) at pH 7.4. The solution of the guest interfering analytes like Cl^-, CH_3COO^-, Br^-, F^-, NO_2^-, $C_2O_4^{2-}$, NO_3^-, SO_4^{2-}, H_2O_2, NB, DNB, 4-NT, 4-NBA, 4-NA, 1,2-CNB, 2,4-DNAN were also prepared in the order of ($c = 2\times10^{-4}$M). Solutions of various concentrations containing sensor and increasing concentrations of analytes were prepared separately. The spectra of these solutions were recorded by means of UV-vis methods.

By fluorescence method:

For fluorescence titrations, stock solution of the sensor ($c = 2 \times 10^{-5}$ M) was prepared for the titration of nitroaromatic compounds (NACs) and anions in CH_3CN-HEPES buffer (7/3, v/v, 25ºC) at pH 7.4. The solution of the guest analytes in the order of 2×10^{-4} M were also prepared. Solutions of various concentrations containing sensor and increasing concentrations of analytes were prepared separately. The spectra of these solutions were recorded by means of fluorescence methods.

Determination of fluorescence quantum yield:

Here, the quantum yield φ was measured by using the following equation,

$$\varphi_x = \varphi_s (F_x / F_s)(A_s / A_x)(n_x^2/ n_s^2)$$

Where,

X & S indicate the unknown and standard solution respectively, φ = quantum yield,

F = area under the emission curve, A = absorbance at the excitation wave length,

n = index of refraction of the solvent. Here φ measurements were performed using anthracene in ethanol as standard [φ = 0.27] (error ~ 10%)

Calculation of the detection limit:

The detection limit DL of **AMN** for PA, NH_3, DNA and BSA was determined from the following equation:

$$DL = K^* Sb_1/S$$

Where K = 2 or 3 (we take 3 in this case); Sb_1 is the standard deviation of the blank solution; S is the slope of the calibration curve.

From the graph Fig.S1, we get slope = 79.096, and Sb_1 value is 231.405

From the graph Fig.S2, we get slope = 344.28, and Sb_1 value is 607.23.

From the graph Fig.S3, we get slope = 184.49, and Sb1 value is 214.39.

From the graph Fig.S4, we get slope = 388.59, and Sb1 value is 66.96.

Thus, using the formula we get the detection limit for PA = 8.77 μM, NH_3 = 5.29 μM, ct DNA = 3.48 μM, BSA = 5.17 μM.

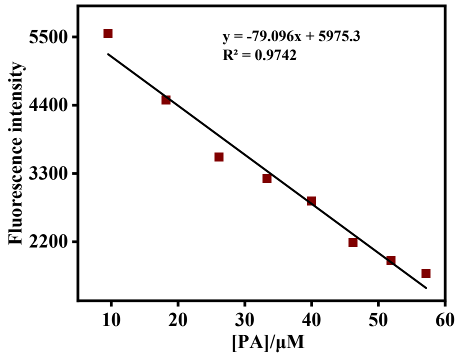

Figure S1: Changes of fluorescence Intensity of **AMN** as a function of PA concentration.

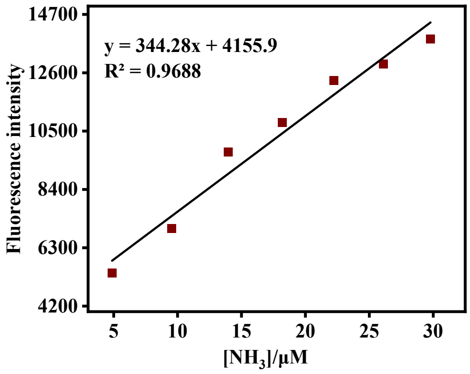

Figure S2: Changes of fluorescence Intensity of **AMN** as a function of NH_3 concentration.

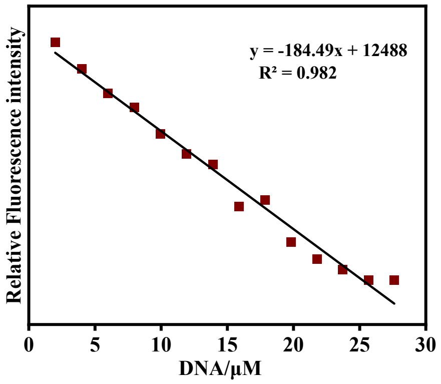

FigureS3: Changes of fluorescence Intensity of **AMN** as a function of ct DNA concentration.

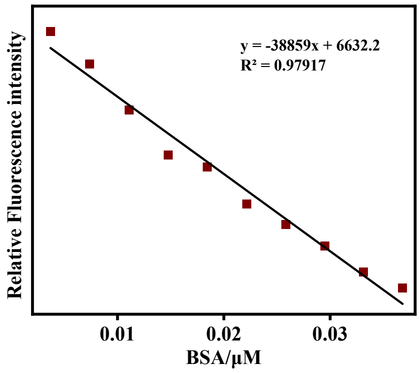

Figure S4: Changes of fluorescence intensity of **AMN** as a function of BSA concentration.

The changes of emission curve of AMN (*c* =2x10^{-5}M) at different time interval by addition of PA, NH_3 and calculation of first order rate constant:

Fig.S5-S8 represents the changes of emission intensity at different time interval by addition of hypochlorite. From the time vs. fluorescent intensity plot at fixed wavelength at 438 nm by using first order rate equation

From fig. S5, we get the rate constant for PA, K = slope × 2.303 = 62.2 × 2.303=143.24 Sec^{-1}.From fig. S6, we get the rate constant for NH_3, K = 143.47× 2.303 = 330.411 Sec^{-1}

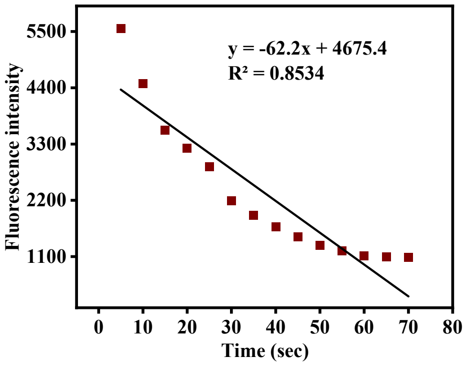

Figure S5: The first order rate equation by using Time vs. fluorescent intensity plot for PA.

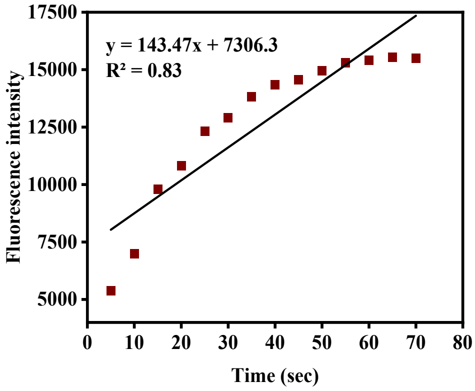

Figure S6: The first order rate equation by using Time vs. fluorescent intensity plot for NH_3.

Stern-Volmer plot

The quenching efficiency and sensitivity were evaluated by calculating the Stern−Volmer quenching constant (K_{SV}), calculated by the Stern−Volmer (SV) equation: $I_0/I = 1 + K_{SV}[COCl_2]$, where K_{SV} is the quenching constant (M^{-1}), I_0 and I are the fluorescence intensities of complexes before and after the addition of NH_3 and PA, and $[NH_3]$ and [PA] is the molar concentration of ammonia and picric acid respectively.

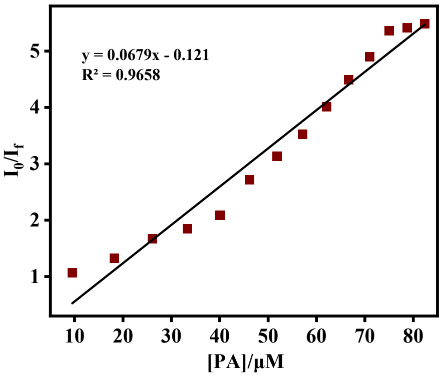

Figure S7: Stern-Volmer plot of **AMN** towards PA.

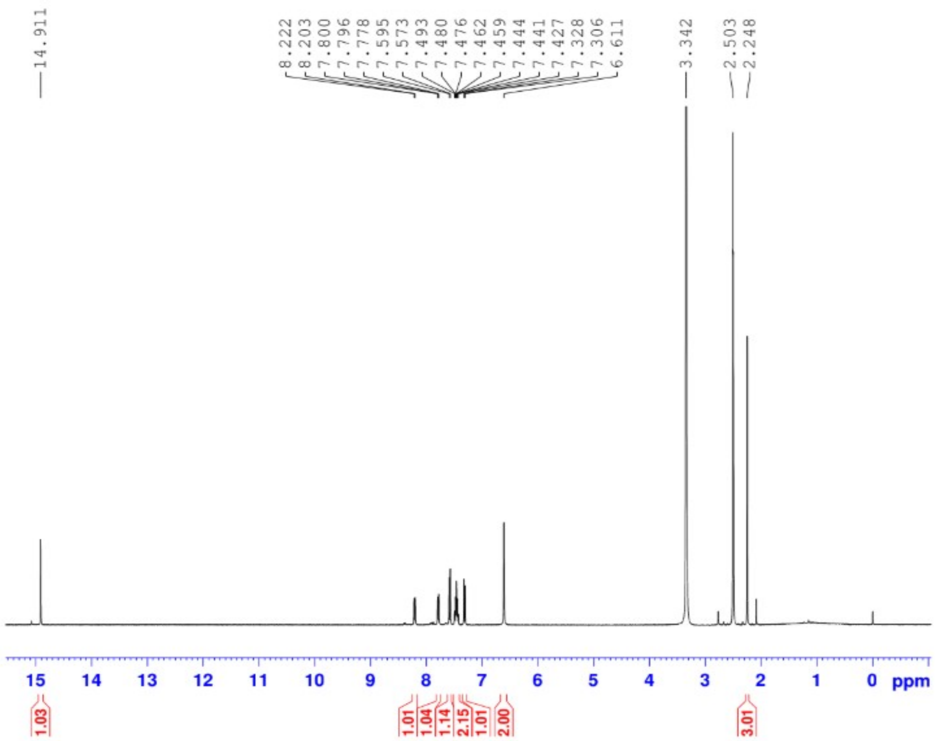

Figure S8: ^{1}H NMR spectrum of **compound 1**

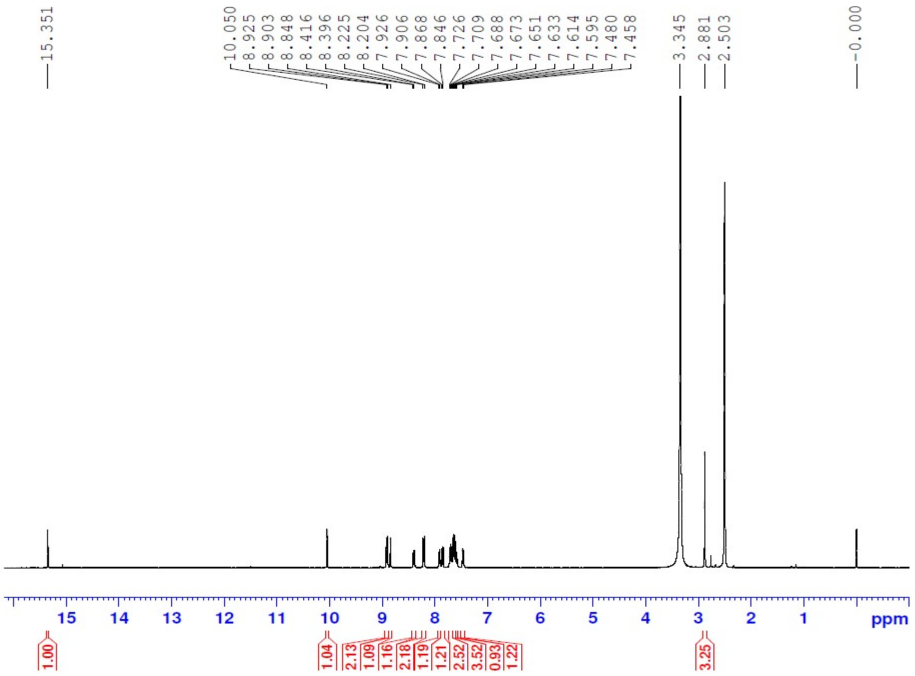

Figure S9: ^{1}H NMR spectrum of **AMN**

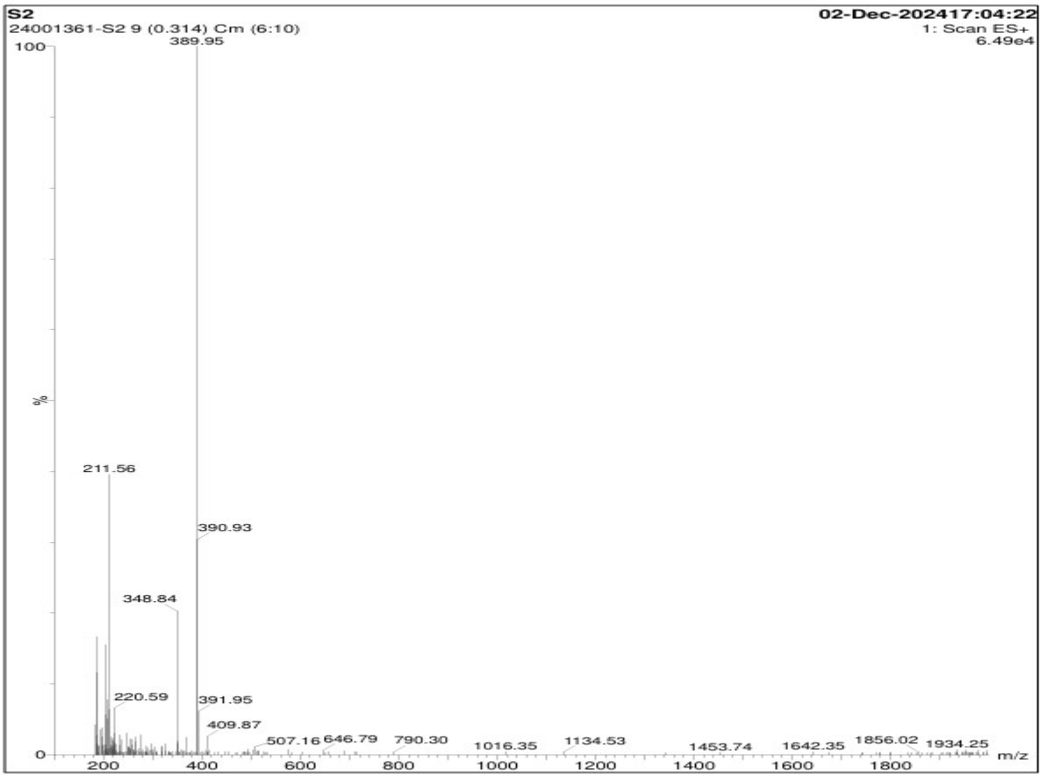

Figure S10: Mass spectrum of **AMN**

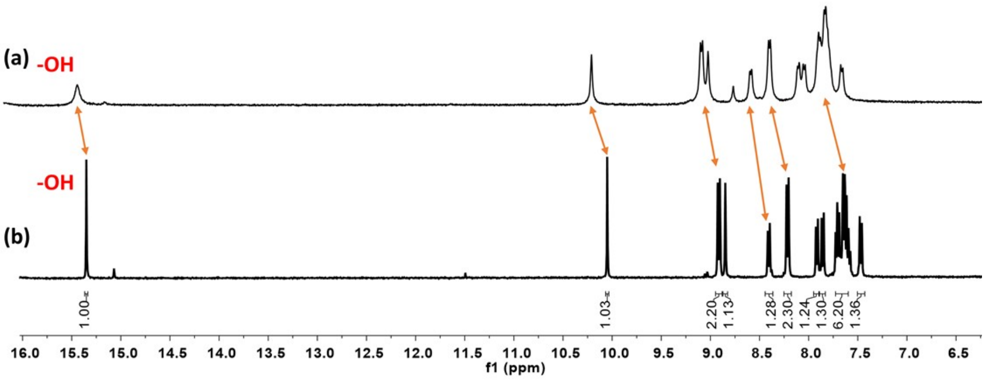

Figure S11: ^{1}H-NMR analysis of (a) **AMN** with picric acid (b) **AMN** in DMSO-d_6.

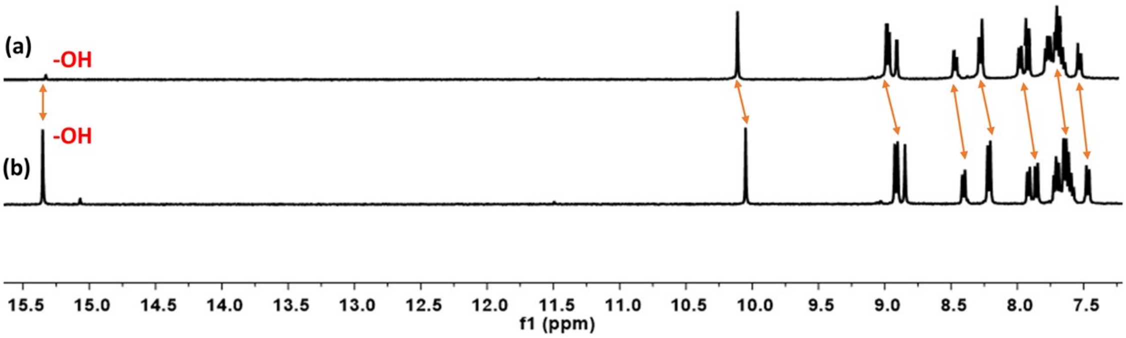

Figure S12: ^{1}H-NMR analysis of (a) **AMN** with NH_3 (b) **AMN** in DMSO-d_6.

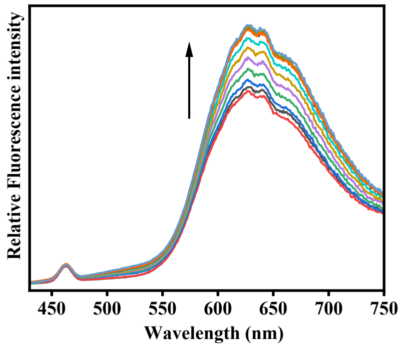

Figure S13: Fluorescence titration of ct DNA-ethiduim bromide system upon addition increment amount of **AMN** (c = 2×10^{-5} M) in Tris-HCl buffer, pH=7.2.

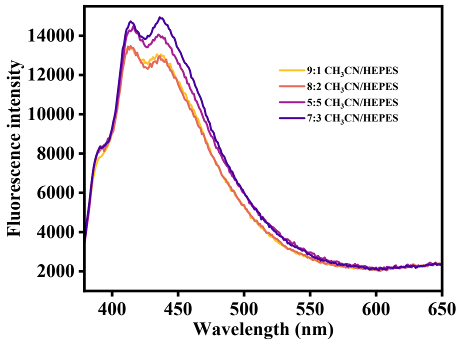

Figure S14: Fluorescent changes of **AMN** (c = 2.0 × 10^{-5} M) with different ratios of CH_3CN/HEPES buffer mixtures.

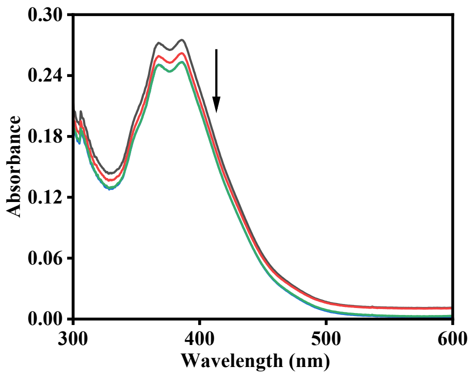

Figure S15: UV-*vis* titration spectra of **AMN** (c = 2.0 × 10^{-5} M) with NH_3 (c =2.0 × 10^{-4} M).

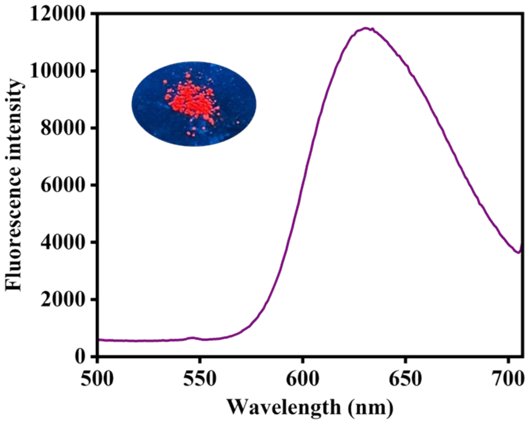

Figure S16: Solid state fluorescence response of **AMN. Inset:** Emission color of **AMN** in solid state under UV lamp.

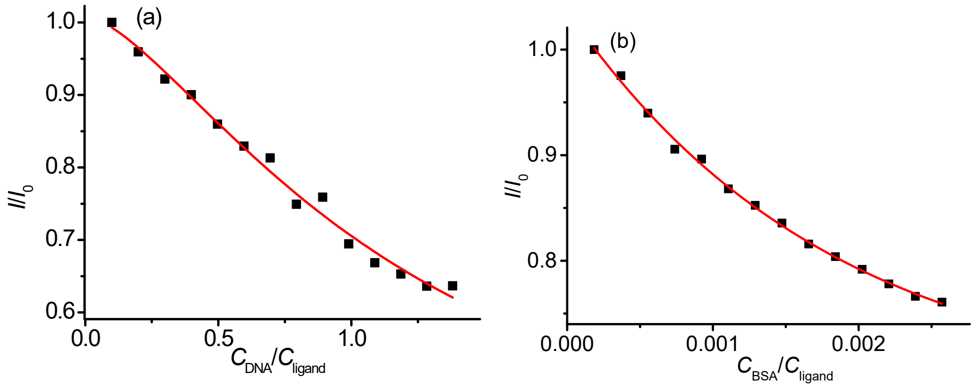

Figure S17: Non-linear fitting curves of binding isotherms resulting from spectrofluorometric titrations of **AMN** (c = 2.0 × 10^{-5} M) with (a) ct DNA and (b) BSA. Red lines represent the best fits to the theoretical model.

The binding constants of **AMN** with ct DNA and BSA were determined from the fluorometric titration spectra by non-linear fitting of the experimental data to the theoretical model in the following equation:[1]

$$\frac{I}{I_0} = 1 + \frac{Q-1}{2}\left(A + xn + 1 - \sqrt{(Q + xn + 1)^2 - 4xn}\right) \quad \text{(Equation 1)}$$

where $Q = I/I_0$ is the minimal emission intensity in the presence of excess ligand; n is the number of independent binding sites.

$A = 1/(K_b \times C_{Lig})$;

$x = C_{\text{ct DNA or BSA}} / C_{Lig}$ is the titration variable

Computational details

Ground state electronic structure calculations in gas phase of the complexes have been carried out using DFT[2] method associated with the conductor-like polarizable continuum model (CPCM).[3] Becke's hybrid function[4] with the Lee-Yang-Parr (LYP) correlation function[5] was used for the study. For H atoms we used 6-31+(g) basis set; for C, N, O atoms we employed LanL2DZ as basis set for all the calculations. The calculated electron-density plots for frontier molecular orbitals were prepared by using Gauss View 5.1 software. All the calculations were performed with the Gaussian 09W software package.[6]

Methods for silico molecular docking studies

The three-dimensional structures of Bovine Serum Albumin (PDB id 4JK4, 2.65 Å, X-ray diffraction) protein and double helical DNA (PDB id 3K5N, 3.15 Å, X-ray diffraction) were downloaded from RCSB PDB (https://www.rcsb.org/). The structures are prepared for docking by using Swiss PDB viewer software. The molecular docking between the ligand and the target molecules (BSA and DNA) were performed by AutoDock Vina software as per our previously published work (Vishnu et al., 2024).[7] There three- and two-dimensional (interaction) rendering of BSA-ligand, and DNA-ligand complexes were performed by UCSF ChimeraX and Biovia Discovery Studio 2024, respectively.

Comparison table of reported sensor for picric acid with this work

Ligand	Analyte	Fluorescence response	LOD	References
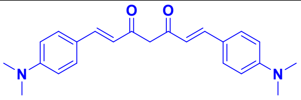	Picric acid	Turn Off	4.21 nM	[8]
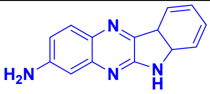	Picric acid	Turn Off	22.74 µM	[9]
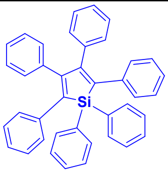	Picric acid	Turn Off	9 nM	[10]
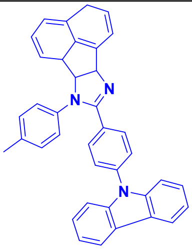	Picric acid	Turn Off	0.853 µM	[11]
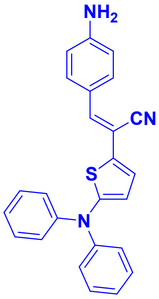	Picric acid	Turn Off	5.7 µM	[12]
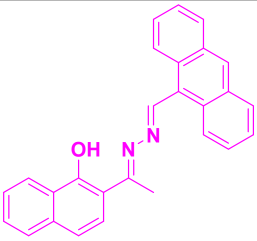	Picric acid	Turn off	8.77 µM	This work

References:

1 F. H. Stootman, D. M. Fisher, A. Rodger, J. R. Aldrich-Wright, Analyst.,2006, 131, 1145.

2. R. G. Parr and W. Yang, *Density Functional Theory of Atoms and Molecules*, Oxford University Press, Oxford, 1989.

3 (*a*) V. Barone and M. Cossi ,*J. Phys. Chem. A,* 1998, **102**, 1995; (*b*) M. Cossi and V. Barone, *J. Chem. Phys.,* 2001, **115**, 4708; (*c*) M. Cossi , N. Rega, G. Scalmani and V. Barone*J. Comp. Chem.,* 2003, **24**, 669.

4 A. D. Becke, *J. Chem. Phys.,* 1993, **98**, 5648.

5 C. Lee , W. Yang and R. G. Parr, *Phys. Rev. B*, 1998, **37**, 785.

6 M. J. Frisch, G. W. Trucks, H. B. Schlegel, G. E. Scuseria, M. A. Robb, J. R. Cheeseman, G. Scalmani, V. Barone, B. Mennucci, G. A. Petersson, H. Nakatsuji, M. Caricato, X. Li, H. P. Hratchian, A. F. Izmaylov, J. Bloino, G. Zheng, J. L. Sonnenberg, M. Hada, M. Ehara, K. Toyota, R. Fukuda, J. Hasegawa, M. Ishida, T. Nakajima, Y. Honda, O. Kitao, H. Nakai, T. Vreven, J. A. Montgomery Jr., J. E. Peralta, F. Ogliaro, M. Bearpark, J. J. Heyd, E. Brothers, K. N. Kudin, V. N. Staroverov, R. Kobayashi, J. Normand, K. Raghavachari, A. Rendell, J. C. Burant, S. S. Iyengar, J. Tomasi, M. Cossi, N. Rega, J. M. Millam, M. Klene, J. E. Knox, J. B. Cross, V. Bakken, C. Adamo, J. Jaramillo, R. Gomperts, R. E. Stratmann, O. Yazyev, A. J. Austin, R. Cammi, C. Pomelli, J. W. Ochterski, R. L. Martin, K. Morokuma, V. G. Zakrzewski, G. A. Voth, P. Salvador, J. J. Dannenberg, S. Dapprich, A. D. Daniels, Ö. Farkas, J. B. Foresman, J. V Ortiz, J. Cioslowski and D. J. Fox, Gaussian Inc., 2009, Wallingford CT.

7 S. Vishnu, A. Nag and A. K. Das, *Anal. Methods*, 2024,16, 5263-5271.

8 Kandasamy Ponnuvel, Govindharasu Banuppriya, Vediappen Padmini,Sensors and Actuators B: Chemical,234,2016, 34-45.

9 Debolina Ghosh, Megha Basak, Deepmoni Deka, Gopal Das, Journal of Molecular Liquids, 363, 2022, 119816.

10 G. He, H. Peng, T. Liu, M. Yang, Y. Zhang and Y. Fang, J. Mater. Chem., 2009, 19, 7347-7353

11 A. B. Kajjam, K. Singh, R. V. Varun Tej and S. Vaidyanathan, Mater. Adv., 2021, 2, 5236-5247

12 X. Lu, G. C. Zhang, D. D. Li, X. H. Tian, W. Ma, S. L. Li, Q. Zhang, H. P. Zhou, J. Y. Wu and Y. P, Dyes Pigm., 2019, 170, 107641.
